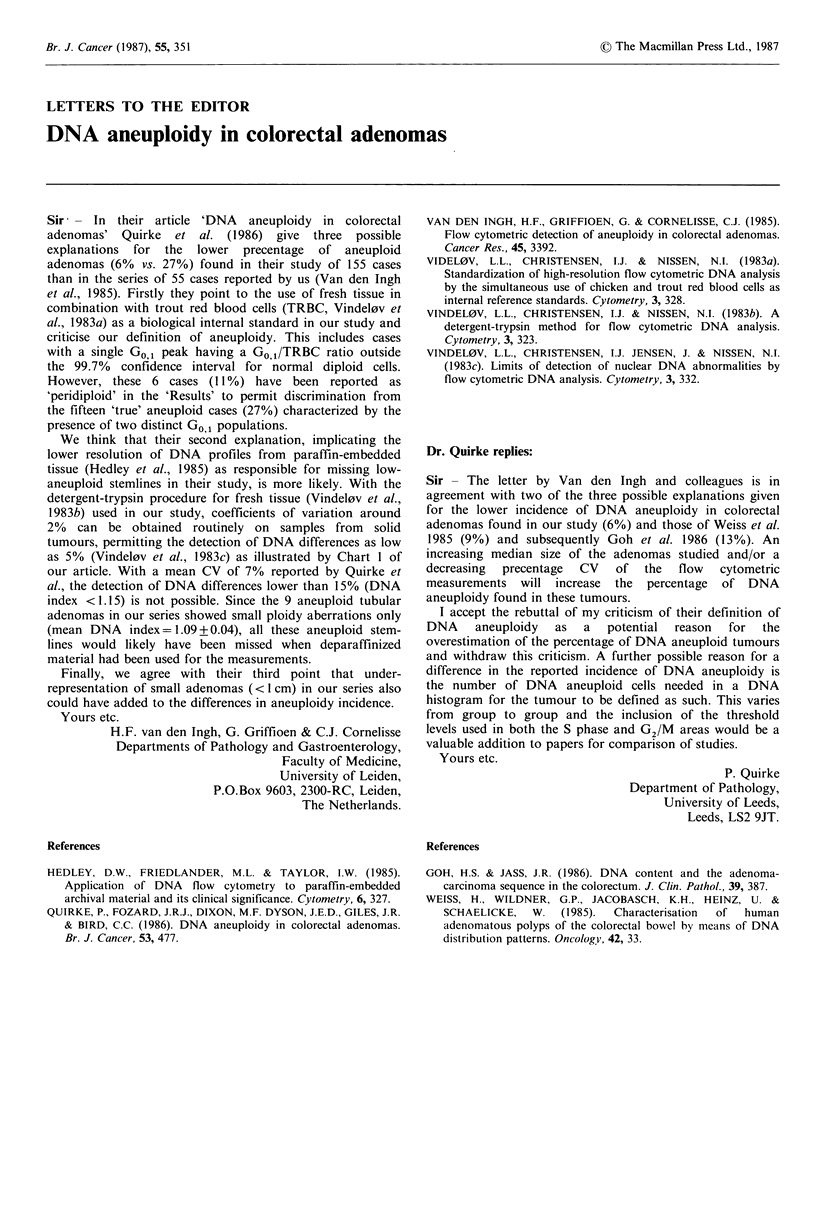# Dr. Quirke replies

**Published:** 1987-03

**Authors:** P. Quirke


### DNA aneuploidy in colorectal adenomas

### Dr. Quirke replies:

**Sir** – The letter by Van den Ingh and colleagues is in agreement with two of the three possible explanations given for the lower incidence of DNA aneuploidy in colorectal adenomas found in our study (6%) and those of Weiss *et al.* 1985 (9%) and subsequently Goh *et al.* 1986 (13%). An increasing median size of the adenomas studied and/or a decreasing precentage CV of the flow cytometric measurements will increase the percentage of DNA aneuploidy found in these tumours.

I accept the rebuttal of my criticism of their definition of DNA aneuploidy as a potential reason for the overestimation of the percentage of DNA aneuploid tumours and withdraw this criticism. A further possible reason for a difference in the reported incidence of DNA aneuploidy is the number of DNA aneuploid cells needed in a DNA histogram for the tumour to be defined as such. This varies from group to group and the inclusion of the threshold levels used in both the S phase and $G_2$/M areas would be a valuable addition to papers for comparison of studies.

Yours etc.

P. Quirke
Department of Pathology,
University of Leeds,
Leeds, LS2 9JT.

### References

GOH, H.S. & JASS, J.R. (1986). DNA content and the adenoma-carcinoma sequence in the colorectum. *J. Clin. Pathol.*, **39**, 387.

WEISS, H., WILDNER, G.P., JACOBASCH, K.H., HEINZ, U. & SCHAELICKE, W. (1985). Characterisation of human adenomatous polyps of the colorectal bowel by means of DNA distribution patterns. *Oncology*, **42**, 33.